# Assessment of changes in the content of anthocyanins, phenolic acids, and antioxidant property of *Saccharomyces cerevisiae* mediated fermented black rice bran

**DOI:** 10.1186/s13568-017-0411-4

**Published:** 2017-06-05

**Authors:** Chaiyavat Chaiyasut, Noppawat Pengkumsri, Sasithorn Sirilun, Sartjin Peerajan, Suchanat Khongtan, Bhagavathi Sundaram Sivamaruthi

**Affiliations:** 1 Innovation Center for Holistic Health, Nutraceuticals and Cosmeceuticals, Faculty of Pharmacy, Chiang Mai University, Chiang Mai, 50200 Thailand; 2 Health Innovation Institute, Chiang Mai, 50230 Thailand

**Keywords:** Anthocyanin, Antioxidant capacity, Fermented rice bran, β-glucosidase, *Saccharomyces cerevisiae*

## Abstract

Studies on phytochemical properties and bioactivities of rice bran revealed the wealth of natural complex antioxidant compounds. The composition and the properties of the rice bran get altered after fermentation by several microbes. This study was designed to optimize the black rice bran fermentation conditions for the total anthocyanin (ACN) content, total antioxidant properties, and relative activity of β-glucosidase (BGS) by *Saccharomyces cerevisiae.* The Box–Behnken design and response surface methodology was employed to achieve the maximum response in fermentation. The kinetic analysis of HPLC based phytochemical determination and bioconversion of ACN, and in vitro antioxidant assays were performed during fermentation. The optimum pH, temperature and NaCl concentration to achieve maximum ACN content, antioxidant capacity, and BGS activity were pH 4.0, 40 °C, and 0.5%, respectively. Bioconversion of cyanidin-3-glucoside and peonidin-3-glucoside to cyanidin and peonidin was recorded at a significant level, respectively. The maximum activity of BGS on rice bran was noticed at 24 h of fermentation. The results suggested that phytochemical content was not changed significantly, whereas the antioxidant properties of rice bran were slightly enhanced after 24 h of fermentation. Additional detailed in vivo evaluation is required to explain the impact of submerged fermentation on the bioactivity of rice bran.

**Electronic supplementary material:**

The online version of this article (doi:10.1186/s13568-017-0411-4) contains supplementary material, which is available to authorized users.

## Introduction

Rice, especially colored or pigmented rice (purple, black, and red rice), is one of the major ration of South East Asian diet (Hu et al. 2003). The phytochemical constituents of pigmented rice are flavonoids, phenolics, tannin, sterols, tocols, *γ*-oryzanols, amino acids, and essential oils (Nakornriab et al. 2008; Min et al. 2009). Rice bran or rice coat is a waste product of agricultural milling process, which is highly enriched with phyto-antioxidant compounds, vitamin, and dietary fibers (Chen et al. 2008; Saenjum et al. 2012). The outcome of research on phytochemical properties and bioactivities of colored rice revealed the richness of natural complex antioxidant compounds, possibly due to the deposition of large amounts of anthocyanin (ACN) (Moldenhauer et al. 2003; Chaudhary 2003).

Anthocyanin are glycosides and belongs to the class of flavonoids (Kong et al. 2003). ACN are reported for its bioactive properties like antioxidant (Pengkumsri et al. 2015), cardiovascular disease prevention (Bell and Gochenaur 2006), anticancer, antitumor, antimutagenic (Marko et al. 2004), antidiabetes (Jayaprakasam et al. 2005), ocular impairment (Canter and Ernst 2004), aging treatments (Lau et al. 2007), and antibacterial activity (Puupponen-Pimia et al. 2005). ACN can be transformed to anthocyanidins (also known as aglycone forms of ACN) by the enzyme called β-glucosidases (BGS) (Fleschhut et al. 2006) and aglycone forms of ACN are more potent than the glycosylated forms (ACN) with respect to antioxidant property (Kahkonen and Heinonen 2003). Cyanidin, delphinidin, peonidin, petunidin, malvidin, and pelargonidin are commonly occurred as aglycone forms of ACN. While in plants, anthocyanidins occur as glycosylated forms, anthocyanins.

β-glucosidase specifically act on β-glucosidic linkages of di- and oligosaccharides, or other glucose conjugates. It comprises a heterogeneous group of enzymes that can cleave the glucosidic linkages and releases glycoside compounds (Hsieh and Graham 2001). BGS are ubiquitous in nature with a vast range of functionality. In bacteria and fungi, BGS are involved in the cellulolytic processes. Studies are representing the presence of BGS in several yeast species like *Hanseniaspora*, *Pichia*, *Candida*, *Saccharomycodes*, *Metschnikowia* and *Brettanomyces,* which are isolated from the grape (Giovanni et al. 2002). *Saccharomyces cerevisiae* is well known for the β-glucosidase activity (Delcroix et al. 1994). BGS can act on ginsenoside, natural steroid glycosides, complex and the hydrolysis of wheat bran ginsenosides by BGS has been reported (Jiang-Ning et al. 2008).

Production of most of the extracellular enzymes by microbes (fungi, yeast and bacteria) are performed using submerged fermentation technique, which provides various advantages including medium sterilization, cultivation of cultures due to easy monitoring and controlling of parameters like nutrients concentration, temperature, aeration, pH and moisture, and end product purification. Submerged fermentation technique had been reported for its use in the extracellular production of BGS by different *Aspergillus* strains (Gunata and Vallier 1999; Jager et al. 2001). Zahoor et al. (2011) reported the use of submerged fermentation technique for the maximum yield of BGS by *Aspergillus niger* in the Eggins and Pugh containing wheat bran (1%). A recent study by Mallek-Fakhfakh et al. (2017) have reported the BGS producing *Talaromyces thermophilus* with the agricultural waste as a substrate using submerged fermentation technique. The current investigation was conceived and the submerged fermentation of black rice bran with BGS producing *S. cerevisiae* was carried out. The impact of three variables such as pH, temperature, and concentration of co-factor (NaCl) on total ACN content and the antioxidant capacity of fermented rice bran have been evaluated. Moreover, the BGS activity during fermentation has also been reported in this study. Optimization and analysis of multiple variables in an experiment can be achieved by response surface methodology (RSM) and Box–Behnken design (BBD) (Woraharn et al. 2015). The basic theoretical and fundamental aspects of RSM have been reported previously (Chandrika and Fereidoon 2005; El-Naggar et al. 2015).

## Materials and methods

### Black rice bran and inoculum preparation

Fresh black rice bran (Chiang Mai province, Thailand) was obtained from rice milling process and sieved through a 60-mesh strainer, and sterilized by autoclave. Then, the sterile bran samples were used for the fermentation process. *S. cerevisiae* HII31 [strain deposited in Thailand Institute of Scientific and Technological Research (TISTR)] cells were grown in yeast extract-peptone-dextrose (YPD) broth [1% yeast extract (Difco Laboratories, Sparks, MD, USA), 2% peptone (Difco Laboratories), 2% glucose] at 30 °C for 48 h. Then the cell density was calculated by spectrophotometer at 600 nm. The cell suspension was adjusted with medium up to 1 × 10^8^ cells/mL (Sirilun et al. 2016).

### Experimental design

Stat-Ease software (Design-Expert 6.0.2, Delaware, USA Echip, 2000-trail version) was used for the experimental design and statistical analysis. A three-factor with three-level of Box–Behnken design was selected to evaluate the effect of the combination of three independent variables such as pH, temperature (°C), and NaCl concentration (%; w/v), coded as X_1_, X_2_, and X_3_, respectively. The values of pH, temperature, and NaCl concentration were set as 3.5–4.5, 35–45 °C, and 0.4–0.6% w/v, respectively (Additional file 1: Table S1). The complete design comprised of 17 experiments including five replicates of the center point. The Z (responses function) was divided into linear, quadratic and interactive values

$$Z = \beta_{0} + \mathop \sum \limits_{i = 1}^{k} B_{i} X_{1} + \mathop \sum \limits_{i = 1}^{k} B_{ii} {\text{X}}^{2} + \mathop \sum \limits_{i > j}^{k} B_{ij} X_{i} X_{j}$$

β_0_, B_i_, B_ii_, and B_ij_ is constant, linear coefficient, quadratic coefficient, and cross-product coefficient, respectively. X_i_ and X_j_ are levels of the independent variables and k represents the number of the tested factors (k = 3) (Fan et al. 2008). RSM was employed to optimize the multiple variants that influence the β-glucosidase activity, anthocyanin content, and antioxidant activity.

### Fermentation

About 25 mL of *S. cerevisiae* suspension (1 × 10^8^ cells/mL) was used for every submerged fermentation process with 25 g of rice bran as a substrate. Fermentation conditions were denoted in Additional file 1: Table S2. The total reaction volume of all the fermentation was maintained as 60 mL, and pH of the mixture was adjusted respectively using 1.0 N HCl before the addition of inoculum. The bio-reaction was performed with constant aeration, and agitation at 150 rpm for 24 h. After 24 h of fermentation, content was separated into two parts like supernatant and bran residue. The supernatant was used for the assessment of β-glucosidase activity, and bran residues were subjected to total anthocyanin extraction subsequently anti-oxidant activity determination.

### Beta-glucosidase activity determination and protein quantification

All the fermented rice bran (FRB) suspension was centrifuged at 5000×*g* for 10 min at 4 °C, and the clear supernatant was collected for the analysis of β-glucosidase activity. The β-glucosidase activity was evaluated by measuring the rate of hydrolysis of *ρ*-nitrophenyl-β-d-glucopyranoside (*ρ*NPG) (Sigma) (Hernandez et al. 2003). In brief, 50 µL of the sample, 100 µL of 3.3 mM *p*-NPG, and 200 µL of 0.5 M Na_2_CO_3_ were mixed. The same mixture without a sample was served as blank. Then, the mixture was incubated at 40 °C for 25 min and measured at 405 nm using a spectrophotometer (DTX880 multimode detector, Beckman Coulter, USA with software version 2.0). The β-glucosidase activity was expressed as relative activity (%). The Lowry’s assay was performed to quantify the protein content in the sample (Lowry et al. 1951). BSA (20–200 µg/mL) has been used for the preparation of standard curve.

### Fermented black rice bran extraction and determination of total anthocyanin

Fermented rice bran samples were extracted with 250 mL of 0.1 N HCl at 40 °C for 30 min. Then the solution was filtrated through 0.45 μm membrane and evaporated under reduced pressure at 40 °C. The recovered crude extracts were kept at −80 °C until use. Total anthocyanin content was determined as described previously (Pengkumsri et al. 2015). Total anthocyanin content was denoted as mg cyanidin chloride equivalent (mg CCE) per gram of FRB.

### Anthocyanins and phenolic acids determination by HPLC

The FRB extracts were used for the determination of the content of anthocyanins and phenolic acids by reversed-phase HPLC as described previously (Pengkumsri et al. 2015). ACE^®^ C18 column (250 mm × 4.6 mm; 5 μm) (Advanced Chromatography Technologies, Scotland) was used for the determination of anthocyanin (detected at a wavelength of 520 nm) and phenolic acids (detected at a wavelength of 280 nm). The mobile phase used for the determination of ACN includes acetonitrile, phosphoric acid (4%) and the rate of flow was set as 1.0 mL/min, and mobile phase used for the determination of phenolic acids includes acetonitrile, trifloroacetic acid (0.1%) and the rate of flow was set as 0.8 mL/min. The gradient elution was performed as described in the previous publication (Pengkumsri et al. 2015). The standards used for the determination of ACN are cyanidin-3-glucoside, peonidin-3-glucoside, cyanidin, and peonidin. The standards used for the determination of phenolic acids are protocatechuic acid, caffeic acid, syringic acid, and p-coumaric acid.

### Antioxidant determination

Antioxidant capacity of FRB extracts were assessed by ABTS (2, 2′-azino-bis-3-ethylbenzthiazoline-6-sulphonic acid), DPPH (1, 1-diphenyl-2-picryl-hydrazil), NO^·^ (Nitric oxide), O_2_^·−^ (superoxide) radical scavenging assay, FRAP (ferric reducing antioxidant power) assay, and inhibition of lipid peroxidation (LPO) assay as described previously (Pengkumsri et al. 2015). The data obtained were expressed as antioxidant activity per gram of FRB.

### Statistical analysis

The 3D response surface plots were showed in this study. Optimized parameters were defined by the Design-Expert software version 6.0.2-trail version and validated through wet lab experiments. The experimental data were established by second-order polynomial regressed equations. Analysis of variance (ANOVA) with a confident interval of 95% (*p* < 0.05) was reported, and all the experiments were performed in triplicates. The statistical program SPSS (v. 17.0) was used for the analysis of significant differences in response to different variables. ANOVA with least significant difference post hoc test was used for determining the significance difference between the respective anthocyanins or anthocyanidins or phenolic acids at different time points of fermentation. Significant difference between the IC_50_ values of the fermented black rice bran in the respective free radical scavenging assay (ABTS or DPPH or superoxide or nitric oxide inhibition assay or inhibition of lipid peroxidation) at 24 h of fermentation and IC_50_ values of fermented black rice bran in the respective free radical scavenging assay at 0 h of fermentation were analyzed by ANOVA with least significant difference post hoc test. The value of p < 0.05 (at confident interval of 95%) was considered significant.

## Results

### Experimental design and validation

The BBD of the current study was tabulated (Additional file 1: Table S2). The pH (X_1_), temperature (X_2_) and NaCl concentration (X_3_) were selected as three independent variables with the range of 3.5–4.5, 35–45 °C and 0.4–0.6%, respectively. A total of 17 different combinations of experiments were generated using design export software v.6. The total ACN content, antioxidant property and relative activity of BGS (%) were the desired outcome (dependent variables) of the current study. ACN content and the antioxidant property have been represented as the mg equivalent of cyanidin/g of FRB and mg TEAC/g of FRB, respectively. All the 17 combinations of experiments were carried out to evaluate the predicted values obtained from the design expert software with respect to all the dependent variables (Table 1). The optimum pH, temperature and NaCl concentration for the maximum recovery of total ACN, BGS activity, and TEAC were tabulated with actual and predicted values (Table 2). In the 17 combinations of experiments, five experiments (run order no. 3, 5, 11, 12, and 16) were with a same combinations of center points (4.0, 40 °C and 0.5%) of three independent variables (pH, temperature and NaCl concentration) that provided the same predicted values. Similarly, the actual values obtained from the five combinations of experiments (run order no. 3, 5, 11, 12, and 16) were found to be not statistically different (Table 1) and therefore, the five combinations of experiments of the center point of three independent variables acts as a biological replicates.

**Table 1:** The predicted and actual values of ACN content, antioxidant capacity of FRB and BGS activity during fermentation

Run order	IV			DV					
	pH	(°C)	NaCl (%)	Total ACN (mg CCE/g FRB)		Antioxidant capacity (mg TEAC/g FRB)		Relative activity (%)	
				Actual	Predicted	Actual	Predicted	Actual	Predicted
1	3.5	45	0.5	78.50	78.15	60.00	59.99	84.08	84.08
2	3.5	35	0.5	82.10	79.70	61.00	60.99	84.34	84.34
3	4.0	40	0.5	100.30^c^	100.88	70.50^b^	70.10	99.90^b^	98.54
4	4.5	40	0.6	95.50	92.78	67.10	67.33	90.70	90.70
5	4.0	40	0.5	100.00^e^	100.88	71.00^a^	70.10	97.20^e^	98.54
6	4.5	45	0.5	82.50	83.05	62.50	62.26	86.13	86.13
7	4.5	40	0.4	91.70	92.78	64.50	64.25	89.56	89.56
8	4.5	35	0.5	83.50	84.60	63.00	63.26	86.39	86.39
9	4.0	45	0.4	90.00	91.15	64.00	63.78	88.83	88.83
10	4.0	35	0.4	91.00	92.70	65.00	65.24	89.09	89.09
11	4.0	40	0.5	100.50^b^	100.88	69.50^d^	70.10	98.10^c^	98.54
12	4.0	40	0.5	104.50^a^	100.88	69.50^d^	70.10	100.00^a^	98.54
13	3.5	40	0.4	86.00	87.88	64.00	64.24	87.51	87.51
14	3.5	40	0.6	87.00	87.88	63.00	63.25	88.65	88.65
15	4.0	35	0.6	93.10	92.70	67.00	66.51	90.23	90.23
16	4.0	40	0.5	100.20^d^	100.88	70.00^c^	70.10	97.50^d^	98.54
17	4.0	45	0.6	92.50	91.15	65.50	65.51	89.97	89.97

*IV* independent variables, *DV* dependent variable, *FRB* fermented rice bran

^a–e^The values of replicates of five combinations of the center points (run order no. 3, 5, 11, 12, and 16) showed no significant difference

**Table 2:** The experimental values and predicted values of responses at the optimum condition

Variables/responses	Optimum conditions	
	Actual value	Predicted value
pH	4.00	4.06
Temperature (°C)	40.00	39.80
NaCl (% w/v)	0.50	0.52
Total anthocyanin content (mg cyanidin equivalent/g of FRB)	101.10 ± 1.91	101.04
Antioxidant capacity TEAC (mg trolox equivalent/g of FRB)	70.10 ± 0.65	70.30
β-glucosidase activity (relative activity (%))	98.54 ± 1.33	98.57

The desirability value of optimum condition was 0.904

*FRB* fermented rice bran

The analysis of variance of the fitted quadratic polynomial model for total ACN content, TEAC, and BGS activity are shown in Additional file 1: Table S3 and the real factors for total ACN, TEAC, and BGS activity were $100.88 + (2.45 \times {\text{pH}}) - (0.77 \times {\text{Temperature}}) - (10.55 \times {\text{pH}}^{2} ) - (8.95 \times {\text{Temperature}}^{2}),$ $70. 10 + \left( { 1. 1 4 \times {\text{pH}}} \right) - \left( {0. 50 \times {\text{Temperature}}} \right) + \left( {0. 6 4 \times {\text{NaCl concentration}}} \right) - \left( { 4. 60 \times {\text{pH}}^{ 2} } \right) - \left( { 3. 8 8 \times {\text{Temperature}}^{ 2} } \right) - \left( {0. 8 5 \times {\text{NaCl concentration}}^{ 2} } \right) + \left( {0. 90 \times {\text{pH}} \times {\text{NaCl concentration}}} \right),$ $9 8. 5 4 + \left( { 1.0 2 \times {\text{pH}}} \right) - \left( {0. 1 3 \times {\text{Temperature}}} \right) + \left( {0. 5 7 \times {\text{NaCl concentration}}} \right) - \left( { 6. 8 6 \times {\text{pH}}^{ 2} } \right) - \left( { 6. 4 4 \times {\text{Temperature}}^{ 2} } \right) - \left( { 2. 5 7 \times {\text{NaCl concentration}}^{ 2} } \right),$ respectively. The reliability of the model has been verified by the determination coefficient (R^2^). The R^2^ value closer to 1 represents the superior correlation between the investigational and predicted values (Jadhav et al. 2013). In this study, we obtained 0.965, 0.9873, and 0.9846 as R^2^ values for total ACN content, TEAC, and BGS activity, respectively. Moreover, the coefficient of variation (CV) was 2.02, 0.79, and 0.92, respectively (Additional file 1: Table S3). The lower value of CV indicates the relatively better reliability of the response model.

The ANOVA and *F* test were performed to evaluate the statistical significance of response surface quadratic model (Table 3). For total ACN content, the linear parameter (X_1_) and quadratic parameters (X_1_^2^ and X_2_^2^) were significant at the level of *p* < 0.01, but the linear parameter (X_2_) was not significant at the level of *p* > 0.05 and did not have interaction parameter power. For TEAC, the linear parameters (X_1_ and X_3_) and quadratic parameters (X_1_^2^, X_2_^2^, and X_3_^2^) were significant at the level of *p* < 0.01, while the linear parameter (X_2_) was significant at the degree of *p* < 0.05. Also, the interaction parameter (X_1_X_3_) was significant at the level of *p* < 0.01.

**Table 3:** Analysis of variance (ANOVA) for the quadratic model of ACN content, TEAC and BGS activity

Source	Degree of freedom	Sum of squares	Mean square	*f* value	*p* value
Total anthocyanin					
Model	4	908.37	227.09	66.01	<0.0001
X_1_	1	48.02	48.02	13.96	0.0028
X_2_	1	4.80	4.80	1.40	0.2602
X_1_^2^	1	470.18	470.18	136.66	<0.0001
X_2_^2^	1	338.41	338.41	98.36	<0.0001
Lack-of-fit	8	26.71	3.44	0.92	0.5776
Pure error	4	14.58	3.64		
TEAC (antioxidant capacity)					
Model	7	186.90	26.70	100.02	<0.0001
X_1_	1	10.35	10.35	38.78	0.0002
X_2_	1	2.00	2.00	7.49	0.0230
X_3_	1	3.25	3.25	12.18	0.0068
X_1_^2^	1	89.09	89.09	333.76	<0.0001
X_2_^2^	1	63.22	63.22	236.84	<0.0001
X_3_^2^	1	3.04	3.04	11.40	0.0082
X_1_X_3_	1	3.24	3.24	12.14	0.0069
Lack-of-fit	5	0.70	0.14	0.33	0.8720
Pure error	4	1.70	0.42		
BGS activity (%)					
Model	6	451.25	75.21	106.65	<0.0001
X_1_	1	8.40	8.40	11.92	0.0062
X_2_	1	0.14	0.14	0.19	0.6708
X_3_	1	2.60	2.60	3.69	0.0838
X_1_^2^	1	198.43	198.43	281.39	<0.0001
X_2_^2^	1	174.63	174.63	247.63	<0.0001
X_3_^2^	1	27.81	27.81	39.44	<0.0001
Lack-of-fit	6	0.000	0.000	0.000	1.0000
Pure error	4	7.05	1.76		

For BGS activity, the linear parameters (X_1_) and quadratic parameters (X_1_^2^, X_2_^2^, and X_3_^2^) were significant at the level of *p* < 0.01, but the linear parameters (X_2_ and X_3_) were not significant at the degree of *p* > 0.05 and did not have interaction parameter power (Table 3).

### Response surface plots

To optimize the desired outcome, the maximum level of responses were predicted by the interaction of two variables with a single constant variable using RSM and BBD. The response surface plots represent the optimum conditions (pH, temperature, and NaCl concentration) for total ACN content, TEAC, and BGS activity, and denoted as cyanidin equivalent, TEAC, and relative activity, respectively.

**Fig. 1:**
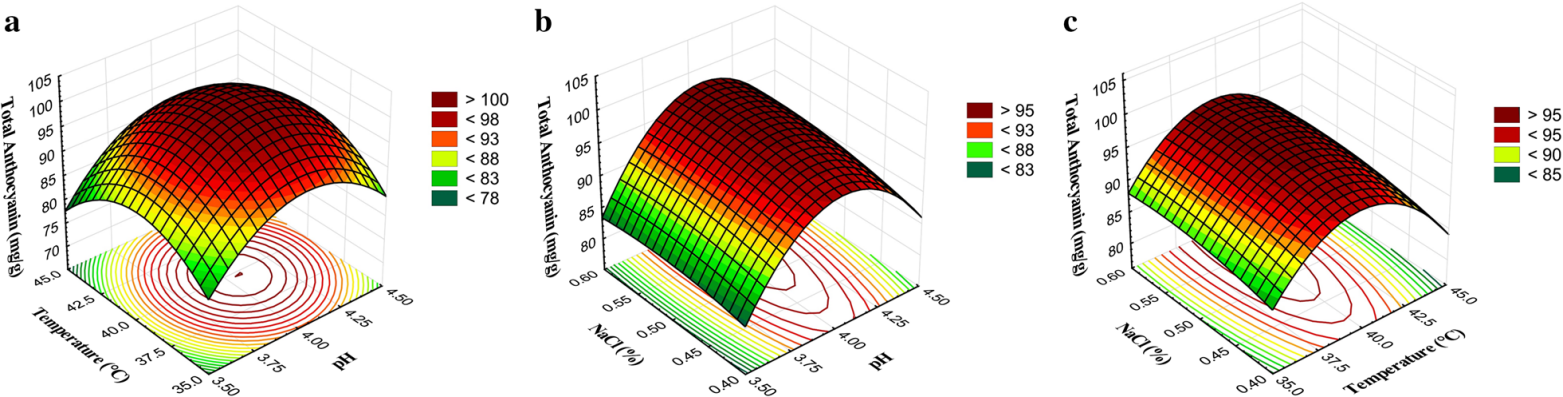
Response surface plot for the total ACN content of FRB describing the interaction of independent variables namely, pH, temperature, and NaCl concentration on ACN content. **a** Effect of temperature and pH on total ACN content of FRB, **b** effect of NaCl concentration (%) and pH on total ACN content of FRB, and **c** effect of NaCl concentration (%) and temperature on total ACN content of FRB

The optimum response of variables for the total ACN content was analyzed and found that the optimum condition was temperature 37.5–42.5 °C and pH 3.75–4.25 with constant cofactor concentration (0.5% NaCl) (Fig. 1a). At constant temperature (40 °C) with variable pH and NaCl concentration, the maximum ACN content was observed in the pH range of 3.75–4.35 (Fig. 1b). Likewise, the interaction of variable temperature and NaCl concentration with constant pH (4.0) condition showed the maximum ACN content at 36.5–43.0 °C (Fig. 1c), and at any case, the concentration of NaCl has no effect on ACN content (Fig. 1).

**Fig. 2:**
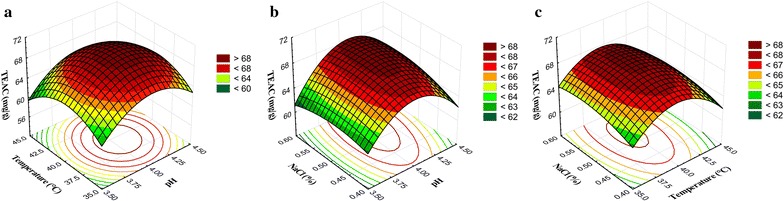
Response surface plot for the antioxidant capacity of FRB describing the interaction of independent variables namely, pH, temperature, and NaCl concentration on antioxidant capacity of FRB. The values were represented as trolox equivalent of antioxidant capacity (TEAC). **a** Effect of temperature and pH on antioxidant capacity of FRB, **b** effect of NaCl concentration (%) and pH on antioxidant capacity of FRB, and **c** effect of NaCl concentration (%) and temperature on antioxidant capacity of FRB

The optimum response of variables for TEAC was also found at 37.5–42.5 °C and pH 3.50–4.25 with constant cofactor concentration (0.5% NaCl) (Fig. 2a). At constant temperature (40 °C) with variable pH and NaCl concentration, the maximum TEAC was observed in the pH range of 3.50–4.25, and 0.43–0.60% of NaCl (Fig. 2b). Similarly, the interaction of variable temperature and NaCl concentration with constant pH (4.0) condition showed the maximum TEAC at 37.0–43.0 °C, and 0.43–0.60% of NaCl (Fig. 2c).

**Fig. 3:**
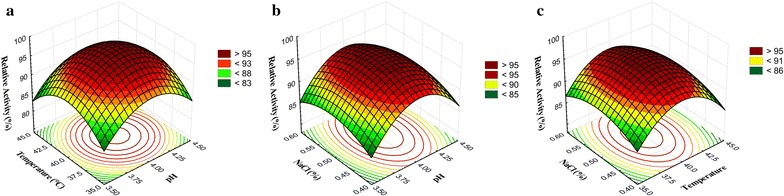
Response surface plot for the β-glucosidase activity during fermentation the interaction of independent variables namely, pH, temperature, and NaCl concentration on β-glucosidase activity during fermentation. The values were represented as relative activity (%). **a** Effect of temperature and pH on enzyme activity of *S. cerevisiae*, **b** effect of NaCl concentration (%) and pH on enzyme activity of *S. cerevisiae*, and **c** effect of NaCl concentration (%) and temperature on enzyme activity of *S. cerevisiae*

The BGS activity at a different temperature, pH, and NaCl concentration was also studied, and the optimum BGS activity was observed at 37.5–42.5 °C, and pH 3.75–4.35 with constant NaCl concentration (0.5%) (Fig. 3a). The optimum BGS activity was observed at the pH of 3.75–4.35 and 0.42–0.60% of NaCl at 40 °C (Fig. 3b). Similarly, the interaction of variable temperature and NaCl concentration with constant pH (4.0) condition represent the maximum BGS activity at 37.0–44.0 °C, and 0.42–0.60% of NaCl (Fig. 3c).

### Anthocyanidins formation

Anthocyanin (cyanidin-3-glucoside and peonidin-3-glucoside) can be transformed to anthocyanidins (cyanidin and peonidin) by the enzyme BGS, which breaks the glycosidic bond of ACN and release the glucose group. The biotransformation of ACN to anthocyanidins during fermentation has been kinetically (0–24 h) evaluated (Additional file 1: Figure S1) and found that the concentration of cyanidin-3-glucoside and peonidin-3-glucoside are gradually decreased from 324.66 ± 16.23 to 190.03 ± 13.25 and 231.65 ± 11.58 to 163.66 ± 10.18 µg/g of FRB, respectively. In the meantime, the amount of cyanidin and peonidin has been increased from 226.73 ± 11.34 to 312.90 ± 11.65 and 86.73 ± 4.43 to 130.93 ± 4.55 µg/g of FRB, respectively. The activity of BGS during fermentation has also been recorded and about 85% of enzyme activity was noticed after 6 h of the process and 100% of relative activity was recorded at 24 h (Table 4). The transformation of ACN to anthocyanidins upon BGS activity was statistically significant (*p* < 0.05) between initial and end points.

**Table 4:** Concentration of anthocyanins (glycoside forms), and anthocyanidins (aglycoside forms) during fermentation and β-glucosidase activity of *S. cerevisiae*

Time (h)	Anthocyanins (µg/g of FRB)		Anthocyanidins (µg/g of FRB)		β-glucosidase [relative activity (%)]
	Cyanidin-3-glucoside	Peonidin-3-glucoside	Cyanidin	Peonidin	
0	324.66 ± 16.23	231.65 ± 11.58	226.73 ± 11.34	86.73 ± 4.34	16.67 ± 2.08
1	319.96 ± 12.76	219.71 ± 10.99	229.74 ± 11.49	94.49 ± 4.72*	41.67 ± 3.15
3	297.43 ± 14.87*	215.74 ± 10.79	244.16 ± 11.21	97.07 ± 3.85*	58.33 ± 3.92
6	275.23 ± 13.76*	188.38 ± 10.42***	258.36 ± 11.92*	114.86 ± 3.74***	85.00 ± 3.25
12	235.09 ± 15.22***	178.49 ± 8.92***	284.06 ± 13.20***	121.29 ± 4.06***	91.67 ± 4.83
18	194.93 ± 12.11***	171.31 ± 9.57***	309.76 ± 10.49***	125.95 ± 4.30***	95.00 ± 3.83
24	190.03 ± 13.25***	163.66 ± 10.18***	312.90 ± 11.65***	130.93 ± 4.55***	100.00 ± 4.00

The significant difference between the anthocyanins (cyanidin-3-glucoside or peonidin-3-glucoside) or anthocyanidins (cyanidin or peonidin) at different time points (1, 3, 6, 12, 18, and 24 h) during fermentation and the respective anthocyanins or respective anthocyanidins at 0 h of fermentation were represented as * (p < 0.05), ** (p < 0.01), and *** (p < 0.001)

### Phenolic acid content

The changes in the representative phenolic acid (protocatechuic acid, caffeic acid, syringic acid, and *p*-coumaric acid) content were assessed during fermentation. The initial amount (at 0 h) of protocatechuic acid, caffeic acid, syringic acid, and *p*-coumaric acid were found as 23.03 ± 1.15, 26.74 ± 1.34, 5.23 ± 0.26, and 195.09 ± 9.75 µg/g of FRB, respectively and after 24 h, the concentration were noted as 22.99 ± 1.15, 27.07 ± 1.35, 5.49 ± 0.27, and 200.55 ± 10.03 µg/g of FRB, respectively (Table 5). Quantification of the phenolic acids was carried out by HPLC and the representative chromatograms was represented in Additional file 1: Figure S2.

**Table 5:** The changes in the content of phenolic acids (µg/g of FRB) during biotransformation

Time (h)	Protocatechuic acid	Caffeic acid	Syringic acid	p-coumaric acid
0	23.03 ± 1.15	26.74 ± 1.34	5.23 ± 0.26	195.09 ± 9.75
1	22.56 ± 1.13	26.05 ± 1.35	5.40 ± 0.27	201.55 ± 10.08
3	21.88 ± 1.09	27.68 ± 1.32	5.00 ± 0.25	185.88 ± 9.95
6	24.00 ± 1.22	25.45 ± 1.26	5.44 ± 0.27	202.32 ± 10.12
12	22.75 ± 1.14	26.87 ± 1.29	5.11 ± 0.28	192.67 ± 9.63
18	23.18 ± 1.16	28.12 ± 1.34	4.97 ± 0.25	204.00 ± 10.10
24	22.99 ± 1.15	27.07 ± 1.35	5.49 ± 0.27	200.55 ± 10.03

Significant difference was not observed between the phenolic acids (protocatechuic acid or caffeic acid or syringic acid or p-coumaric acid) at different time points (1, 3, 6, 12, 18, and 24 h) during fermentation and the respective phenolic acids at 0 h of fermentation

### Antioxidant properties

**Fig. 4:**
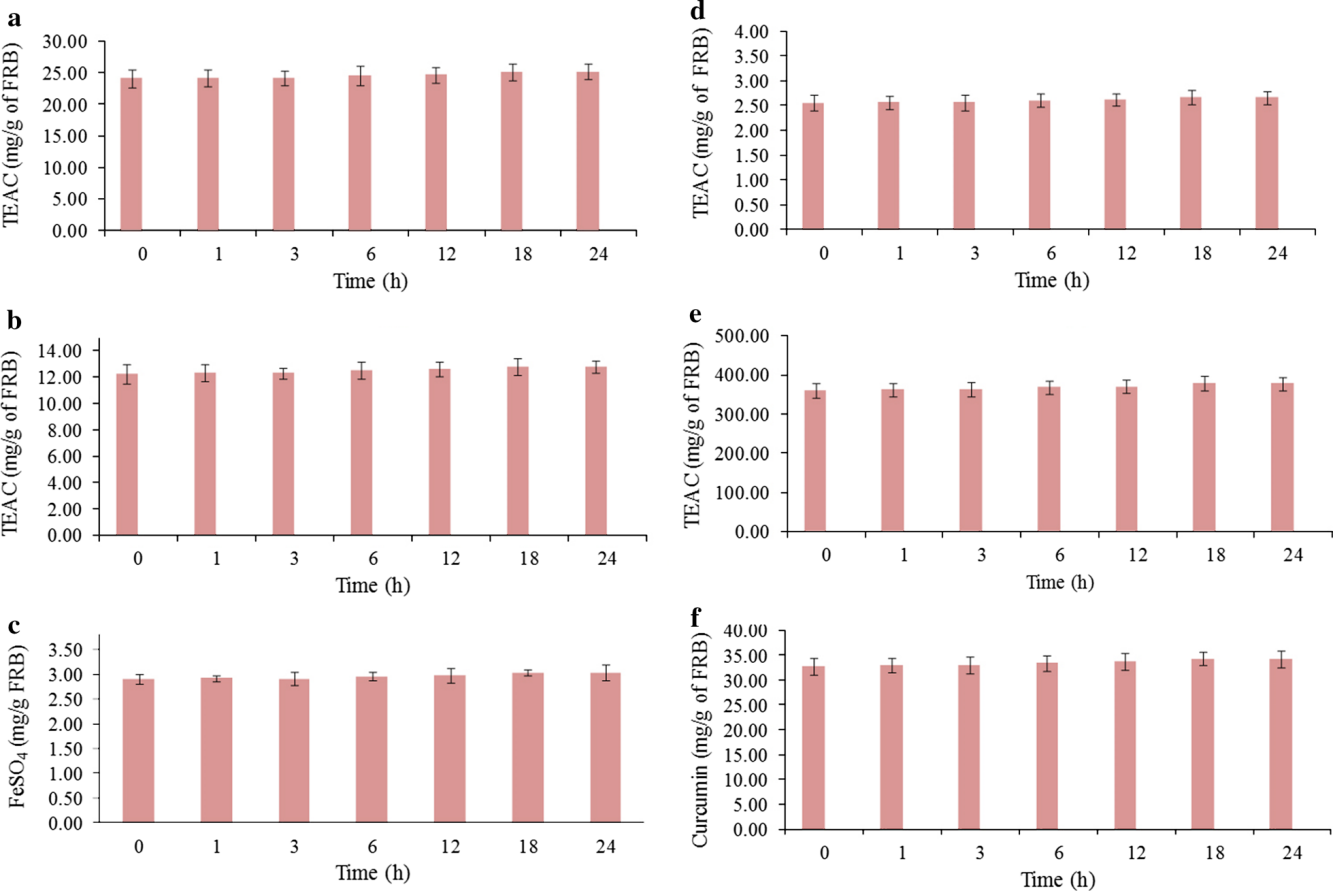
Assessment of free radical scavenging activity during *S. cerevisiae* mediated fermentation of rice bran. **a** ABTS assay, **b** DPPH assay, **c** FRAP assay, **d** inhibition of lipid peroxidation, **e** superoxide and **f** nitric oxide radical scavenging assays for fermented rice bran. **a** ABTS assay, **b** DPPH assay, **d** inhibition of lipid peroxidation, **e** superoxide radical scavenging assays results were represented as trolox equivalent of antioxidant capacity (TEAC). FRAP assay and nitric oxide scavenging assay results were denoted as FeSO_4_ equivalent/g of FRB and curcumin equivalent/g of FRB, respectively

The antioxidant property of RB before and after the fermentation with BGS producing *S. cerevisiae* has been kinetically evaluated. The representative antioxidant assays such as ABTS, DPPH, inhibition of lipid peroxidation, superoxide, nitric oxide, and FRAP assays were performed. ABTS, DPPH, inhibition of lipid peroxidation, and superoxide assay results were represented as trolox equivalent of antioxidant capacity (mg/g of FRB), whereas the results of nitric oxide and FRAP assays were denoted as curcumin equivalent and FeSO_4_ equivalent (mg/g of FRB), respectively. The changes in the antioxidant properties were represented in Fig. 4.

The ABTS, DPPH, inhibition of lipid peroxidation, superoxide, nitric oxide, and FRAP assay results suggested that the free radical scavenging property of RB was not significantly altered after the fermentation (24 h), when compared with that at the 0 h of fermentation. The free radical inhibition ability of FRB has been represented as an IC_50_ (concentration of FRB that are capable of exhibiting 50% of inhibition of free radical) concentration for respective free radical assays. The IC_50_ values were not significantly altered after fermentation (Table 6).

**Table 6:** The free radical inhibition activities of fermented black rice bran

Time (h)	ABTS assay (µg of FRB)	DPPH assay (mg of FRB)	Inhibition of lipid peroxidation assay (mg of FRB)	Superoxide assay (µg of FRB)	Nitric oxide assay (mg of FRB)
0	183.75 ± 7.19	1.06 ± 0.03	7.25 ± 0.32	88.72 ± 3.44	1.24 ± 0.07
1	182.81 ± 9.14	1.06 ± 0.05	7.22 ± 0.36	88.26 ± 4.41	1.24 ± 0.06
3	183.05 ± 7.15	1.06 ± 0.02	7.23 ± 0.30	88.38 ± 5.42	1.24 ± 0.08
6	180.07 ± 9.00	1.04 ± 0.05	7.11 ± 0.36	86.94 ± 4.35	1.22 ± 0.06
12	178.83 ± 8.94	1.04 ± 0.05	7.06 ± 0.35	86.34 ± 3.32	1.21 ± 0.05
18	175.70 ± 7.78	1.02 ± 0.04	6.94 ± 0.33	84.83 ± 4.24	1.19 ± 0.05
24	175.72 ± 8.79	1.02 ± 0.05	6.94 ± 0.33	84.84 ± 3.24	1.19 ± 0.06

The values are IC_50_ concentrations of FRB in the respective analysis

Significant difference was not observed between the IC_50_ values of fermented black rice bran in the respective free radical scavenging assay (ABTS or DPPH or lipid peroxidation or superoxide or nitric oxide inhibition assay) at 24 h of fermentation and IC_50_ values of fermented black rice bran in the respective free radical scavenging assay at 0 h of fermentation

## Discussion

To study the influence of fermentation conditions on ACN content, antioxidant property (denoted as trolox equivalent of antioxidant capacity; TEAC) and BGS activity during black rice bran fermentation by *S. cerevisiae*, BBD and surface response methodology was employed. Extremely low probability values of the model for all decided responses (Total ACN, TEAC, and BGS activity) revealed that the current model is reliable and significant (*p* < 0.0001). The lack of fit values is typically used to measure the failure of the model generated by the software. This value should not be non-significant (Sharma et al. 2009). It is essential to validate the fitted model to confirm the created approximate values with wet lab results. The proposed model should be with acceptable fit value, if not the results are not reliable (Murugesan et al. 2007). The lack of fit values of total ACN, TEAC, and BGS activity (*p* value = 0.5776, 0.8720, and 1.000, respectively) suggested that the experimental model was statistically significant.

The response surface plots were represented to show the influence of independent variables on desired outcomes (Figs. 1, 2 and 3), which revealed the optimum conditions to achieve maximum anticipated products (total ACN content, TEAC, and BGS activity). Avila et al. (2009) reported about bioconversion of ACN by *Lactobacillus* (*L. acidophilus*, *L. plantarum*, and *L. casei*) and *Bifidobacterium* (*B. lactis*) with the involvement of BGS activity. These strains can release the metabolites like gallic, syringic and homogentisic acids by the enzymatic degradation of malvidin (an O-methylated anthocyanidin) and the authors claimed that *B. lactis*, *L. casei*, and *L. plantarum* strains are functional probiotics to acquire additional malvidin and phenolic acids (Avila et al. 2009). The results of the current study suggested that the BGS of *S. cerevisiae* also can stimulate the bioconversion of ACN, and the rate of conversion was directly proportional to the enzyme activity, and the maximum bioconversion rate was observed after 24 h of fermentation (Table 4).

*Lactobacillus* spp. mediated fermentation decreases the content of most of the polyphenols of red sorghum, whereas the antibiotic nature of the sorghum was not affected (Svensson et al. 2010). Ryan et al. (2011) demonstrated that *Saccharomyces boulardii* mediated fermentation altered the phytochemical nature of RB and also fermented RB showed the improved ability to suppress the growth of human B lymphomas. The phenolic content of RB was doubled after fermentation by *Rhizopus oryzae* and showed higher inhibition potential in DPPH and peroxidase assays (Schmidt et al. 2014). The results of the present study suggested that phenolic content of RB was not significantly altered after fermentation indicating that phytochemical nature of RB was not influenced by the BGS activity of *S. cerevisiae*.

The bioactivity of the anthocyanins also depends on the nature and location of the glycosidic groups, especially influences the antioxidant activity (Avila et al. 2009). Thus, the bioconversion slightly modifies the free radical scavenging activity of ACN. After fermentation, the IC_50_ values were not significantly changed (Table 6), which suggested that *S. cerevisiae* mediated fermentation process slightly increases the quality of the rice bran. Tsuda et al. (1994) reported the antioxidant activity of ACN pigments, and results suggested that cyanidin had stronger bioactivity than cyanidin 3-O-β-glucoside, and α-tocopherol especially in lipid peroxidation inhibition assay. The bioactivities of aglycons and glycosides are not fluctuated and influenced by emulsion, whereas the aglycons showed high low-density lipoprotein reduction activity than the glycosides (Kahkonen and Heinonen 2003). In the current study also, improved (statistically significant) bioactivity has been observed after fermentation, which suggested that the aglycone form of rice phytochemical are more potent than glycone form.

The optimum conditions for the ACN content, antioxidant activity and relative activity of BGS with RB as a substrate using RSM and BBD has been revealed. The optimum pH, temperature, and NaCl concentration for RB fermentation was 4, 40 °C, and 0.5%, respectively with respect to the ACN content, TEAC, and BGS activity. The bioconversion of ACN by BGS, phytochemicals, and free radical scavenging activity of fermented RB has been demonstrated. The results revealed that fermentation of RB with BGS producing *S. cerevisiae* HII31 enhanced the bioactivity of RB, significantly. Further, in vivo evaluation of bioactivity of FRB are required to explain the impact of fermentation on the ability of RB bioactivity.

## Additional file

**Additional file 1: Figure S1.** Representative Chromatograms showed the variation in ACN content of RB during fermentation. (a) ACN standards (1. Cyanidin-3-glucoside, 2. Peonidin-3-glucoside, 3. Cyanidin, 4. Peonidin), (b) 0 h sample (c) 12 h fermented samples (d) 24 h fermented samples. **Figure S2.** Representative Chromatograms showed the variation in phenolic acid content of RB during fermentation. (a) Phenolic acid standards (1. Protocatechuic acid, 2. Caffeic acid 3. Syringic acid, 4. *p*-coumaric acid), (b) 0 h sample (c) 12 h fermented samples (d) 24 h fermented samples. **Table S1.** The optimization code of independent variables and actual values. **Table S2.** The Box-Behnken design for anthocyanin content, antioxidant capacity of black RB, and β-glucosidase activity during fermentation. X_1,_ X_2,_ X_3_ is pH, temperature (°C), and NaCl concentration (%), respectively. **Table S3.** Codes and process variable levels used in experimental design.

## Abbreviations

ACN: anthocyanin
BGS: β-glucosidase
HPLC: high-performance liquid chromatography
RSM: response surface methodology
BBD: Box–Behnken design
YPD: yeast extract-peptone-dextrose
FRB: fermented rice bran
CCE: cyanidin chloride equivalent
ABTS: 2, 2′-azino-bis-3-ethylbenzthiazoline-6-sulphonic acid
DPPH: 1, 1-diphenyl-2-picryl-hydrazil
FRAP: ferric reducing antioxidant power
LPO: lipid peroxidation
ANOVA: analysis of variance
TEAC: trolox equivalent of antioxidant capacity
R^2^: determination coefficient
CV: coefficient of variation
*S. cerevisiae*: *Saccharomyces cerevisiae*
*L. acidophilus*: *Lactobacillus acidophilus*
*L. plantarum*: *Lactobacillus plantarum*
*L. casei*: *Lactobacillus casei*
*B. lactis*: *Bifidobacterium lacis*
*S. boulardii*: *Saccharomyces boulardii*
