# Supplementary material for: Assessment of changes in the content of anthocyanins, phenolic acids, and antioxidant property of Saccharomyces cerevisiae mediated fermented black rice bran
Source: AMB Express. 2017 Jun 5;7:114. doi: 10.1186/s13568-017-0411-4 (PMC5459781; doi:10.1186/s13568-017-0411-4)
Supplement: Supplementary file 1 — Additional file 1: Figure S1. Representative Chromatograms showed the variation in ACN content of RB during fermentation. (a) ACN standards (1. Cyanidin-3-glucoside, 2. Peonidin-3-glucoside, 3. Cyanidin, 4. Peonidin), (b) 0 h sample (c) 12 h fermented samples (d) 24 h fermented samples. Figure S2. Representative Chromatograms showed the variation in phenolic acid content of RB during fermentation. (a) Phenolic acid standards (1. Protocatechuic acid, 2. Caffeic acid 3. Syringic acid, 4. p-coumaric acid), (b) 0 h sample (c) 12 h fermented samples (d) 24 h fermented samples. Table S1. The optimization code of independent variables and actual values. Table S2. The Box-Behnken design for anthocyanin content, antioxidant capacity of black RB, and β-glucosidase activity during fermentation. X1, X2, X3 is pH, temperature (°C), and NaCl concentration (%), respectively. Table S3. Codes and process variable levels used in experimental design. [file 13568_2017_411_MOESM1_ESM.docx]

**Assessment of changes in the content of anthocyanins, phenolic acids, and antioxidant property of *Saccharomyces cerevisiae* mediated fermented black rice bran**

Chaiyavat Chaiyasut^1^, Noppawat Pengkumsri^1^, Sasithorn Sirilun^1^, Sartjin Peerajan^2^, Suchanat Khongtan^2^, Bhagavathi Sundaram Sivamaruthi^1^*

^1^Innovation center for Holistic Health, Nutraceuticals and Cosmeceuticals, Faculty of Pharmacy, Chiang Mai University, Chiang Mai 50200, Thailand. Emails: [chaiyavat@gmail.com](mailto:chaiyavat@gmail.com) (CC); [p_arkasus@hotmail.com](mailto:p_arkasus@hotmail.com) (NP); [ssirilun@gmail.com](mailto:ssirilun@gmail.com) (SS)

^2^ Health Innovation Institute, Chiang Mai 50230, Thailand. Email: [sartjin_p@yahoo.com](mailto:sartjin_p@yahoo.com) (SP); [farfon1993@hotmail.com](mailto:farfon1993@hotmail.com) (SK)

*Correspondence: [sivasgene@gmail.com](mailto:sivasgene@gmail.com), [sivamaruthi.b@cmu.ac.th](mailto:sivamaruthi.b@cmu.ac.th); Tel.: +6653944341. Fax: +6653894163.

**Table S1. The optimization code of independent variables and actual values**

| **Independent variable** | **Symbol** | **Code levels** | | |
| --- | --- | --- | --- | --- |
|  |  | **-1** | **0** | **1** |
| pH | X_1_ | 3.5 | 4.0 | 4.5 |
| Temperature (°C) | X_2_ | 35 | 40 | 45 |
| Sodium chloride (%, w/v) | X_3_ | 0.4 | 0.5 | 0.6 |

**Table S2.** **The Box-Behnken design for anthocyanin content, antioxidant capacity of black RB, and β-glucosidase activity during fermentation. X_1,_ X_2,_ X_3_ is pH, temperature (°C), and NaCl concentration (%), respectively.**

| **Run order** | **Codes** | | | **Experimental value** | | |
| --- | --- | --- | --- | --- | --- | --- |
|  | **X_1_** | **X_2_** | **X_3_** | **X_1_** | **X_2_** | **X_3_** |
| 1 | -1 | 1 | 0 | 3.5 | 45 | 0.5 |
| 2 | -1 | -1 | 0 | 3.5 | 35 | 0.5 |
| 3 | 0 | 0 | 0 | 4.0 | 40 | 0.5 |
| 4 | 1 | 0 | 1 | 4.5 | 40 | 0.6 |
| 5 | 0 | 0 | 0 | 4.0 | 40 | 0.5 |
| 6 | 1 | 1 | 0 | 4.5 | 45 | 0.5 |
| 7 | 1 | 0 | -1 | 4.5 | 40 | 0.4 |
| 8 | 1 | -1 | 0 | 4.5 | 35 | 0.5 |
| 9 | 0 | 1 | -1 | 4.0 | 45 | 0.4 |
| 10 | 0 | -1 | -1 | 4.0 | 35 | 0.4 |
| 11 | 0 | 0 | 0 | 4.0 | 40 | 0.5 |
| 12 | 0 | 0 | 0 | 4.0 | 40 | 0.5 |
| 13 | -1 | 0 | -1 | 3.5 | 40 | 0.4 |
| 14 | -1 | 0 | 1 | 3.5 | 40 | 0.6 |
| 15 | 0 | -1 | 1 | 4.0 | 35 | 0.6 |
| 16 | 0 | 0 | 0 | 4.0 | 40 | 0.5 |
| 17 | 0 | 1 | 1 | 4.0 | 45 | 0.6 |

**Table S3. Codes and process variable levels used in experimental design**

| **Responses** | **Quadratic polynomial model equations** | **R^2^** | **CV (%)** |
| --- | --- | --- | --- |
| Total anthocyanin | 100.88 + 2.45X_1_ - 0.77X_2_ - 10.55X_1_^2^ - 8.95X_2_^2^ | 0.9565 | 2.02 |
| Antioxidant capacity | 70.10 + 1.14X_1_ - 0.50X_2_ + 0.64X_3_ - 4.60X_1_^2^ - 3.88X_2_^2^-0.85X_3_^2^ + 0.90X_1_X_3_ | 0.9873 | 0.79 |
| Relative activity (%) | 98.54 + 1.02X_1_ - 0.13X_2_ + 0.57X_3_ - 6.86X_1_^2^ - 6.44X_2_^2^ - 2.57X_3_^2^ | 0.9846 | 0.92 |

Note: CV is coefficient of variation; X_1_, pH; X_2_, temperature (°C); X_3_, NaCl concentration (% w/v).

**Supplementary figure: 1**

**
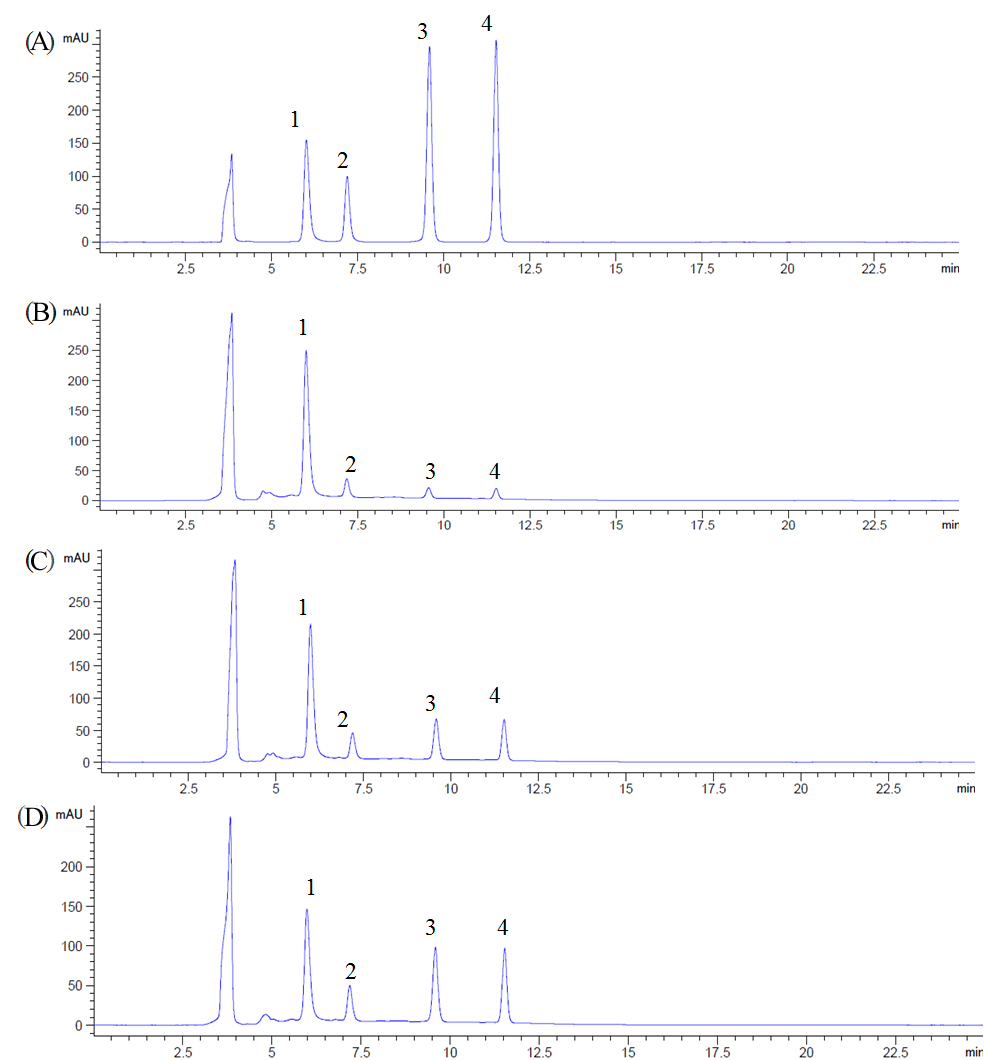
**

**Fig. S1** Representative Chromatograms showed the variation in ACN content of RB during fermentation. (a) ACN standards (1. Cyanidin-3-glucoside, 2. Peonidin-3-glucoside, 3. Cyanidin, 4. Peonidin), (b) 0 h sample (c) 12 h fermented samples (d) 24 h fermented samples.

**Supplementary figure: 2**


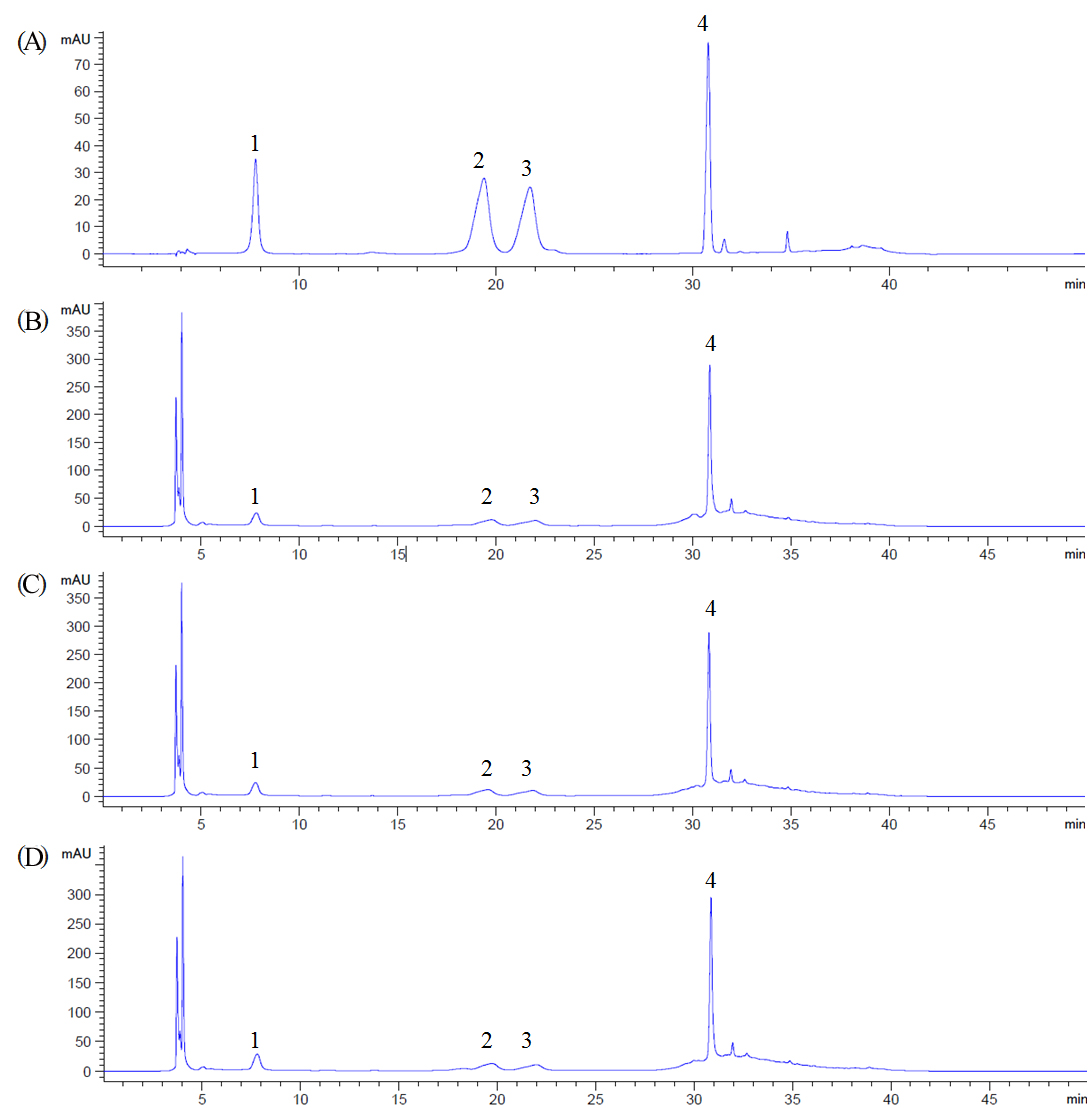


**Fig. S2** Representative Chromatograms showed the variation in phenolic acid content of RB during fermentation. (a) Phenolic acid standards (1. Protocatechuic acid, 2. Caffeic acid 3. Syringic acid, 4. *p*-coumaric acid), (b) 0 h sample (c) 12 h fermented samples (d) 24 h fermented samples.
